# Use of Sensitive, Broad-Spectrum Molecular Assays and Human Airway Epithelium Cultures for Detection of Respiratory Pathogens

**DOI:** 10.1371/journal.pone.0032582

**Published:** 2012-03-05

**Authors:** Krzysztof Pyrc, Karol Stożek, Krzysztof Wojcik, Katarzyna Gawron, Slawomir Zeglen, Wojciech Karolak, Jacek Wojarski, Marek Ochman, Magdalena Hubalewska-Mazgaj, Grazyna Bochenek, Marek Sanak, Marian Zembala, Andrzej Szczeklik, Jan Potempa

**Affiliations:** 1 Microbiology Department, Faculty of Biochemistry Biophysics and Biotechnology, Jagiellonian University, Krakow, Poland; 2 Division of Cell Biophysics, Faculty of Biochemistry Biophysics and Biotechnology, Jagiellonian University, Krakow, Poland; 3 Department of Cardiac Surgery and Transplantology, Silesian Center for Heart Diseases, Zabrze, Poland; 4 Department of Medicine, Jagiellonian University Medical College, Krakow, Poland; 5 Oral Health and Systemic Disease Research Group, School of Dentistry, University of Louisville, Louisville, Kentucky, United States of America; University of Kansas Medical Center, United States of America

## Abstract

Rapid and accurate detection and identification of viruses causing respiratory tract infections is important for patient care and disease control. Despite the fact that several assays are available, identification of an etiological agent is not possible in ∼30% of patients suffering from respiratory tract diseases. Therefore, the aim of the current study was to develop a diagnostic set for the detection of respiratory viruses with sensitivity as low as 1–10 copies per reaction. Evaluation of the assay using a training clinical sample set showed that viral nucleic acids were identified in ∼76% of cases. To improve assay performance and facilitate the identification of novel species or emerging strains, cultures of fully differentiated human airway epithelium were used to pre-amplify infectious viruses. This additional step resulted in the detection of pathogens in all samples tested. Based on these results it can be hypothesized that the lack of an etiological agent in some clinical samples, both reported previously and observed in the present study, may result not only from the presence of unknown viral species, but also from imperfections in the detection methods used.

## Introduction

Respiratory infections are an important cause of morbidity and mortality, with a worldwide disease burden estimated at almost 98,000,000 disability adjusted life years (DALYs) and more than 4,000,000 deaths per year [1], [2]. Several studies show that respiratory infections have the most prominent impact on the pediatric population, and it is assumed that preschool children experience 6–10 viral infections per year, while older children, adolescents and adults experience 3–5 per year [3], [4], [5]. Clinical presentation of viral respiratory infections depends heavily on patient status and the pathogen involved, and may show a broad variety of symptoms, including both upper (e.g. common cold, rhinitis, sinusitis, otitis media) and lower respiratory symptoms (e.g. pneumonia, bronchopneumonia, bronchitis, bronchiolitis, tracheitis and croup). Rapid and accurate detection and identification of viruses causing respiratory tract infections is important for patient care and disease control as this significantly decreases the length of hospitalization and reduces management costs. Furthermore, detection and identification of etiological agents enables the introduction of specific antiviral treatments and appropriate isolation precautions in severe cases [6], [7]. Despite this, no etiological agent can be identified in ∼30% of patients suffering from respiratory tract diseases. Previous studies hypothesized that this might be due to the presence of as yet unknown viruses [8], [9], [10], [11], [12].

Virus detection and typing is a tedious task because the presence of multiple viral species and strains blurs the image, and infection with a wide range of pathogens may result in a similar clinical outcome [13], [14], [15], [16], [17], [18], [19]. Numerous clinical virology laboratories continue to employ diagnostic algorithms, which incorporate antigen or culture-based methods. The latter have several drawbacks in that they are technically demanding and laborious. Furthermore, not all viruses replicate in cell culture (e.g. human bocavirus and human coronavirus HKU1) and the panel of susceptible cell lines is limited [20], [21], [22]. The undeniable advantage of this approach is that the recovered viral strains are valuable for further pathogen characterization and antiviral susceptibility testing [23], [24]. Antigen-based methods can be used for both detection and typing of pathogens in clinical samples or in cell culture. Until now, several assays have been developed, although high variability of viruses may pose a problem and lead to false-negative results [17], [25], [26]. Indisputably, the most sensitive and specific methods for detection of respiratory viruses are molecular techniques [17], [27], [28], [29].

Molecular methods may be roughly divided into sensitive and specific assays such as real-time PCR or loop-mediated isothermal amplification (LAMP) methods, and techniques with broad specificity that are capable of detecting a wide variety of pathogens. The latter group includes sequence-independent methods (e.g. virus discovery based on cDNA-AFLP (VIDISCA), Sequence-independent, single-primer amplification (SISPA), differential display) [23], [30], [31], [32], [33]. The main disadvantage of such an approach is that the sensitivity of the assays is limited. Further, these methods are relatively laborious and high-throughput analysis is unworkable. Some previously developed methods (e.g. universal primers or microarray-based methods) are designed to detect a broad range of targets combine, at least partially, the advantages of these two approaches.

In the present study, human ciliated airway epithelial cell cultures were used [34], [35], [36], [37], [38] in combination with a newly developed set of nested PCR assays with broad specificity, which are able to detect all known strains of respiratory viruses while disregarding co-infections, genetic variability, and other material contained in the clinical sample. Assay design was based on all available sequence data for human respiratory viruses; therefore, one may assume that they may enable the detection of previously uncharacterized viral strains. The combination of this highly sensitive culture system with a set of optimized molecular assays should allow improved detection in clinical samples. This hypothesis was verified using a set of clinical samples from patients suffering from respiratory disease.

## Results

### Primer design and nested PCR

To design the sensitive and specific assays, all available sequences from public databases were analyzed. Primer design was based on sequence analysis facilitating the identification of conserved regions within viral genomes. Primer blasting showed no significant similarity to human genomic sequences and primer pairs with the best thermodynamic parameters were chosen for subsequent studies. The selected primer pairs are listed in **Table 1**. Primers used for the detection of rhinoviruses have been previously described and were found to be superior to any other assay [39].

**Table 1:** Sequences of primers used for nested-PCR detection of respiratory pathogens.

Virus group	Reaction	Primer name	Primer sequence^a^	Product length^b^
Adenoviruses	1^st^ PCR	Adeno_F1	TTCCCCATGGC**B**CACAACAC	481 bp
		Adeno_R1	CCCTGGTAGCC**R**AT**R**TTGTA	
	2^nd^ PCR	Adeno_F2	TCCAT**Y**CC**H**TC**K**CGCAACTGGGC	302 bp
		Adeno_R2	GAACCAGTC**Y**TTGGTCATGTT	
Influenza A	1^st^ PCR	INF1_5a	CAAT**Y**GAGGAGTGCCTGATTAA	92 bp
		INF1_3a	GCC**HY**A**R**CTAT**Y**T**Y**A**R**TGCATGT	
	2^nd^ PCR	INF2_5a	CCTGATTAA**Y**GATCCCTGGG	64 bp
		INF2_3a	**Y**A**R**TGCATGT**K**Y**N**AGGAAGGAG	
Influenza B	1^st^ PCR	INFB_F1	GTCTGTTTCCAAAGATCAAAGGC	460 bp
		INFB_R1	ATGGCTTC**R**TACCCAACCATAGAG	
	2^nd^ PCR	INFB_F2	TCG**R**TTTATAGGAAGAGCAATGGC	177 bp
		INFB_R2	TAG**R**TCTTC**W**ATGTCTGCAATCCC	
Enteroviruses	1^st^ PCR	5enter_pcr1	TCCGGCCCCTGAATGCGGCTAATC	187 bp
		3enter_pcr1	TGGCCAATCCAATAGCTATATGG	
	2^nd^ PCR	5enter_pcr2	CCCTGAATGCGGCTAATCC**Y**AAC	178 bp
		3enter_pcr2	CCAATCCAATAGCTATATGG**Y**AAC	
Respiratory syncytial virus	1^st^ PCR	hRSVFa1	CTCAGTGTAGGTAGAATGTTTGC	450 bp
		hRSVRa1	GATATCT**R**TATAATCCACTTTGTTCATC	
	2^nd^ PCR	hRSVFa2	T**K**ACAAGATATGGTGATCTAGA	204 bp
		hRSVRa2	TGTAC**W**CCATGCAGTTCATC	
Alphacoronaviruses	1^st^ PCR	CoV1F1	TACAGGGTCCTCCTGGTAGTG	461 bp
		CoV1R1	G**K**CCTATAGCACACATACGTTG	
	2^nd^ PCR	CoV1F2	G**R**GTTGAGTGTTATAGTGG	240 bp
		CoV1R2	GG**Y**TCCATAACACCTTTAG	
Betacoronaviruses	1^st^ PCR	CoV2F1	TATTATGTTAAGCCTGGTGG	268 bp
		CoV2R1	CCATCATCACTCAAAATCATC	
	2^nd^ PCR	CoV2F2	ACTAG**Y**AGTGGTGATGCA	244 bp
		CoV2R2	TCATCACTCAAAATCATCATAC	
Bocavirus	1^st^ PCR	BoV_5a	GGCAAT**W**CTGT**W**TCTCATGTTCA	236 bp
		BoV_3a	CTGTGAATG**W**GTAGGACAAAGG	
	2^nd^ PCR	BoV_5b	CAGCTAAATTTTATGAAACAACT	160 bp
		BoV_3b	CCAAGAGGAAATGAGTTTGGAA	
Parainfluenza 1	1^st^ PCR	PI_5a	TTCTGGAGATGTCCCGTAGG	294 bp
		PI_3a	TCCTGTTGTCGTTGATGTCA	
	2^nd^ PCR	PI_5b	CC**YY**TACTGAGCAACAACCC	265 bp
		PI_3b	TTGTCGTTGATGTCATAGGT	
Parainfluenza 2	1^st^ PCR	PII_Fa	CGAACTGCCACAATTCTTGG	420 bp
		PII_Ra	ATGTTGCTGAGGGGATAAAGC	
	2^nd^ PCR	PII_Fb	CTATGTTCAAGTATTCTTCATGA	272 bp
		PII_Rb	ATCATGCAGAAGCAGATTTCC	
Parainfluenza 3	1^st^ PCR	PIII_Fa	AACTGTAAACTCAGACTTGGTAC	345 bp
		PIII_Ra	TATTGGATGTTCAAGACCTCC	
	2^nd^ PCR	PIII_Fb	GACTTGGTACCTGACTTAAATCC	278 bp
		PIII_Rb	TATCCCTGGTCCAACAGATGG	
Human metapneumovirus	1^st^ PCR	hMPV_F1	AA**R**GAAAGAGAATTAAGTGTAGG	405 bp
		hMPV_R1	TTGTTTCTGGTGGTGCATGTC	
	2^nd^ PCR	hMPV_F2	GAAAC**Y**TTAACAAAGTATGGTG	172 bp
		hMPV_R2	GCTGT**R**GTTTCATATCTAAAGGC	

a degenerated positions are marked with bold;

b expected size calculated for a single strain.

### Identification of viruses in cell culture supernatants and clinical samples

All primer sets developed *in silico* were evaluated in an experimental set-up. Briefly, primer sets were tested on positive control material and the resulting products were analyzed on agarose gels. Several optimization steps allowed the selection of PCR conditions common for all reactions, including primer concentration, amount of template, and PCR profile.

**Figure 1:**
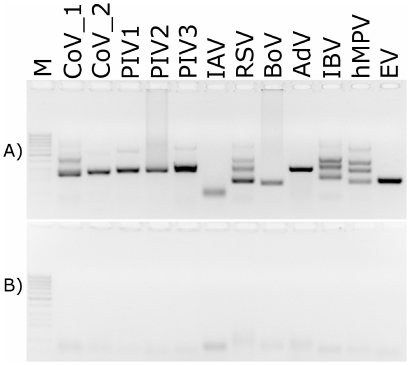
Amplification of viral nucleic acids using the developed primer sets; A) Positive samples (plasmids containing template for PCR amplification) B) Negative controls. M: size marker (GeneRuler 50–1000 bp DNA; Fermentas); CoV_1: HCoV-NL63, CoV_2: HCoV-HKU1; RSV: respiratory syncytial virus; IAV: influenza A virus; PIV: parainfluenza virus type 1, 2 or 3; BoV: bocavirus; AdV: adenovirus type 4; IBV: influenza B virus; hMPV: human metapneumovirus; EV: echovirus 9. Analysis was performed on 1.5% agarose gel.

To test assay performance, a set of plasmids containing genetic elements recognized by certain primers was prepared. All plasmids were used as an input for nested PCR, and the resulting products were analyzed on agarose gels. All reactions resulted in the appearance of specific bands (**Figure 1**).

**Figure 2:**
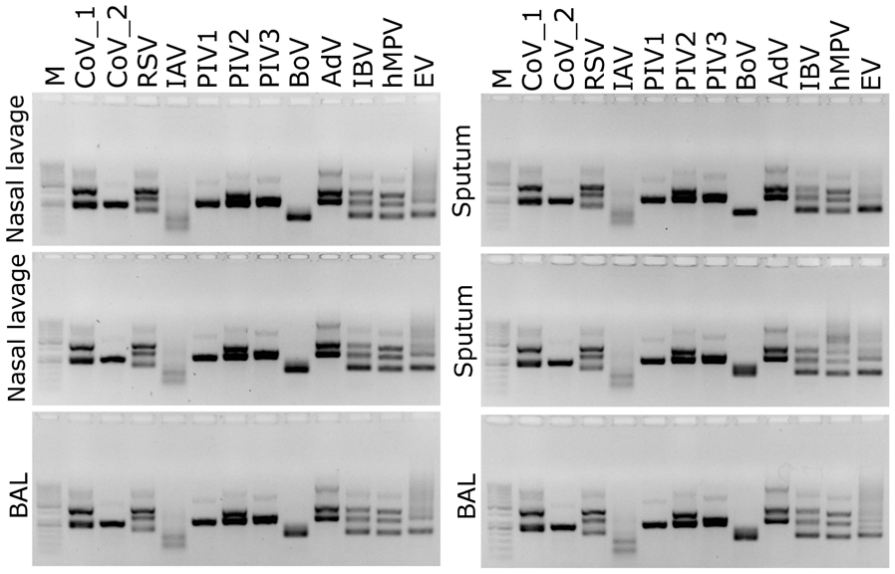
Detection of respiratory viral pathogens in different clinical materials (nasal lavages, sputum, and bronchioalveolar lavage (BAL)). M: size marker (GeneRuler 50–1000 bp DNA; Fermentas); CoV_1: HCoV-NL63, CoV_2: HCoV-HKU1; RSV: respiratory syncytial virus; IAV: influenza A virus; PIV: parainfluenza virus type 1, 2 or 3; BoV: bocavirus; AdV: adenovirus type 4; IBV: influenza B virus; hMPV: human metapneumovirus; EV: echovirus 9. Analysis was performed on 1.5% agarose gel.

To show whether it is possible to detect viral pathogens in clinical material, a set of virus culture supernatants, or clinical isolates, was prepared, which contained HCoV-NL63, HCoV-229E, HCoV-HKU1, RSV, influenza A and B viruses, parainfluenza 1, 2, 3 viruses, human bocavirus, human adenovirus, human metapneumovirus or human respiratory enterovirus. Virus titers were determined for all cultivable pathogens, and samples were diluted to a TCID_50_ per mL of 1. For pathogens that were not cultured, samples were diluted 10 times and used as an input for amplification. Selected clinical samples from patients not presenting with obvious clinical symptoms of respiratory disease or tested negative for respiratory viruses were used as dilution media. The resulting PCR products were visualized on agarose gels and are shown in **Figure 2**. Clearly, all positive samples yielded bands corresponding in size to the predicted products. No inhibition of the PCR reaction was observed in clinical samples and no amplification was detected in the negative controls. Sequencing confirmed the identity of the PCR products.

### Assay sensitivity

**Figure 3:**
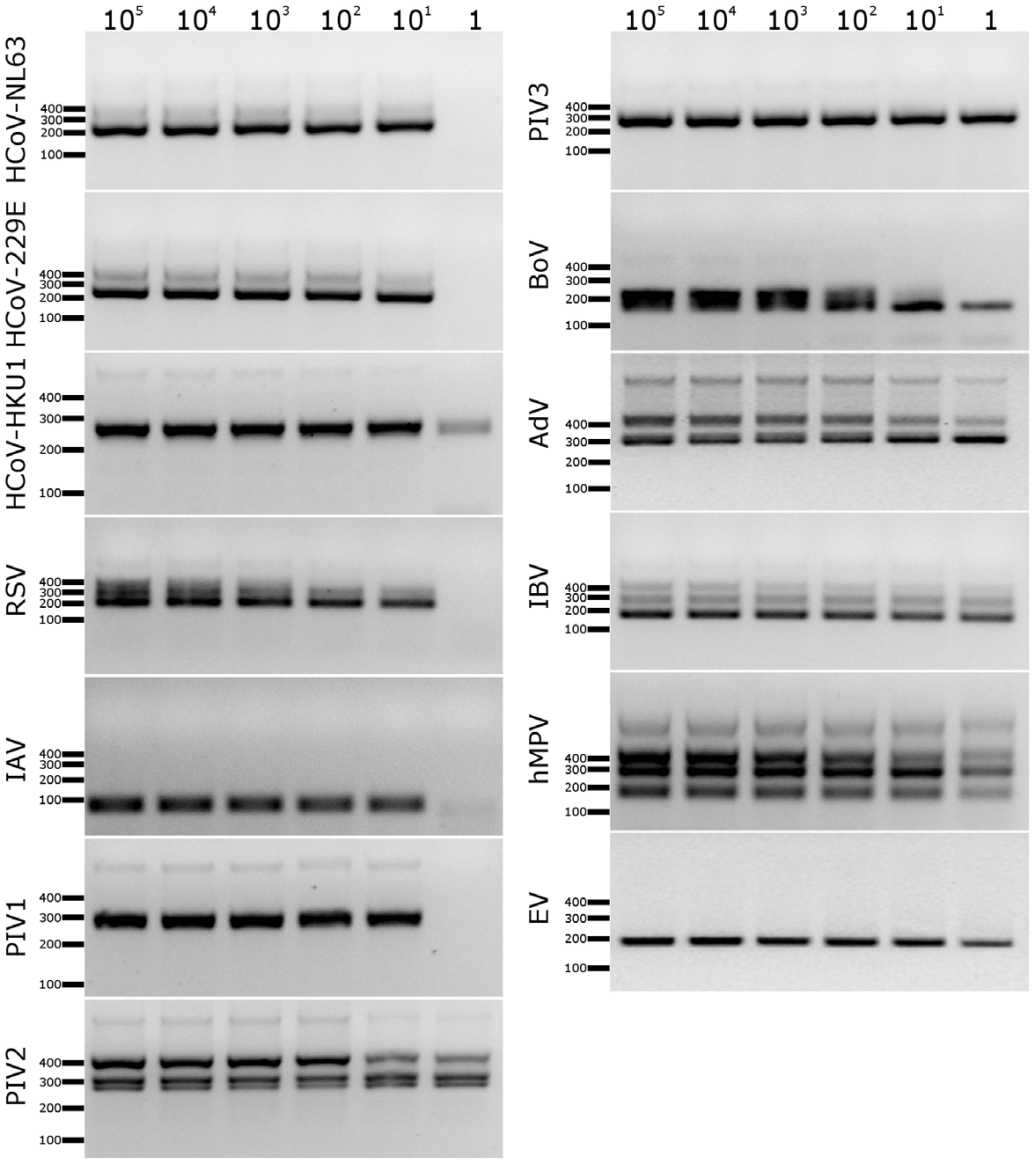
Sensitivity of the developed assays. Numbers at the top of the figure denote virus copy number per reaction. CoV: coronavirus; RSV: respiratory syncytial virus; IAV: influenza A virus; PIV: parainfluenza virus type 1, 2 or 3; BoV: bocavirus; AdV: adenovirus type 4; IBV: influenza B virus; hMPV: human metapneumovirus; EV: echovirus 9. GeneRuler 50–1000 bp DNA (Fermentas) marker was used as a size marker. Analysis was performed on 1.5% agarose gel.

Serial dilutions of control plasmids containing genetic elements recognized by the PCR primers were used to determine the sensitivity of each assay. Briefly, after assessment of plasmid concentration using two independent methods (gel electrophoresis and spectrophotometrically), the plasmid copy number was set to 10^5^ copies/µl and serially diluted to 10^0^ copies/µl. One microliter of such a template was further used as an input for the PCR reaction. The results presented in **Figure 3** show that the assay sensitivity varied from 1 to 10 copies of template nucleic acids per reaction. Careful optimization showed that the assay sensitivity could be further increased. By increasing the primer concentration in the first PCR to 600 nM, a detection level of 1 copy per reaction was reached. Unfortunately at this sensitivity the image becomes blurred and difficult to interpret due to the appearance of non-specific products. No such distortion was seen in the clinical samples, most likely because of the presence of nucleic acids originating from eukaryotic cells that non-specifically bind to dome of the primers (data not shown).

### Cross-reactivity

**Figure 4:**
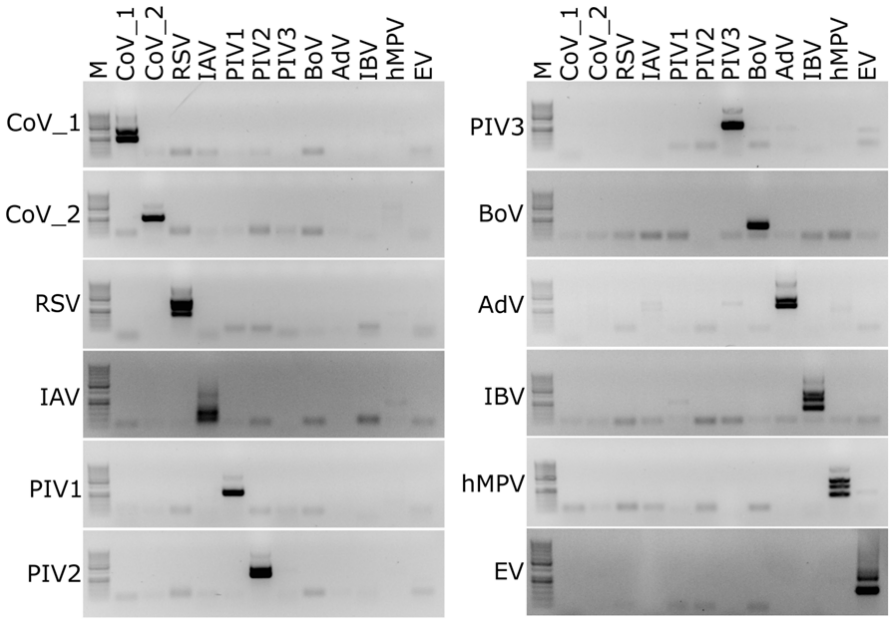
Specificity of the developed assays. All primer pairs (names on top of the figure) were used to amplify all virus stocks included in the study (names on the left side of each panel). M: size marker (GeneRuler 50–1000 bp DNA; Fermentas); CoV_1: HCoV-NL63, CoV_2: HCoV-HKU1; RSV: respiratory syncytial virus; IAV: influenza A virus; PIV: parainfluenza virus type 1, 2 or 3; BoV: bocavirus; AdV: adenovirus type 4; IBV: influenza B virus; hMPV: human metapneumovirus; EV: echovirus 9. Analysis was performed on 1.5% agarose gel.

To determine whether the newly designed assays were specific for the selected pathogen, all primer pairs were tested for cross-reactivity against all other respiratory pathogens included in the study. Briefly, RNA or DNA was isolated from concentrated cell culture samples or clinical specimens and subjected to reverse transcription, where appropriate. The resulting cDNA was used as an input for nested PCR using all the primer sets shown in **Table 1**. No cross-reactivity was detected in any of the assays, confirming high specificity of the PCR reactions (**Figure 4**).

### Detection of viruses in clinical samples

**Figure 5:**
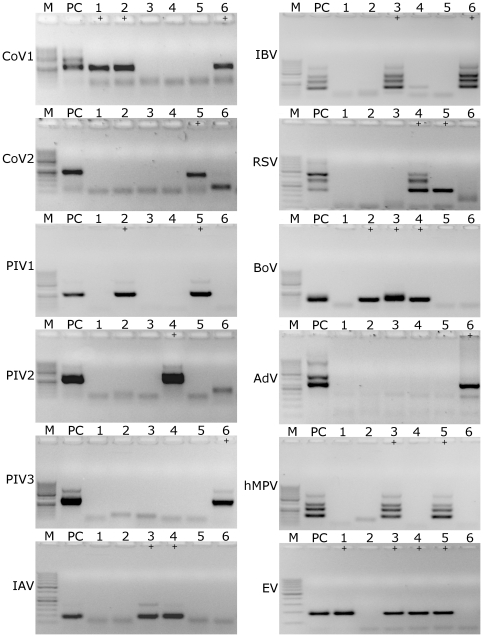
Detection of viruses in clinical material. Detection of respiratory viral pathogens in clinical specimens (nasal lavages). M: size marker (GeneRuler 50–1000 bp DNA; Fermentas); PC: positive control; Clinical samples are denoted with consecutive numbers. Samples considered to be positive are marked with “+” sign. CoV1: alphacoronavirus, CoV2: betacoronavirus; RSV: respiratory syncytial virus; IAV: influenza A virus; PIV: parainfluenza virus type 1, 2 or 3; BoV: bocavirus; AdV: adenovirus; IBV: influenza B virus; hMPV: human metapneumovirus; EV: enterovirus. Analysis was performed on 1.5% agarose gel.

A total of 34 samples were subjected to analysis. Briefly, RNA or DNA was isolated and samples processed as described in “Materials and Methods”. Further, samples were used as an input for the nested PCR developed within the current study. The results are presented in **Table 2** and show that an etiological agent was identified in ∼76% of cases, and the frequency of occurrence of certain species was consistent with the available literature data [40]. Furthermore, two or more pathogens were identified in the same sample in ∼65% of cases (**Table 3** **,** **Figure 5**).

**Table 2:** Detection of respiratory viruses in clinical samples.

Pathogen	Cases [no (%)]; PCR^a^	Cases [no (%)]; HAE+PCR^b^
alphacoronavirus	1 (2.94%)	4 (11.76%)
betacoronavirus	2 (5.88%)	3 (8.82%)
RSV	0 (0.00%)	0 (0.00%)
influenza A	6 (17.65%)	6 (17.65%)
influenza B	2 (5.88%)	2 (5.88%)
parainfluenza 1	1 (2.94%)	2 (5.88%)
parainfluenza 2	0 (0.00%)	2 (5.88%)
parainfluenza 3	3 (8.82%)	3 (8.82%)
bocavirus	0 (0.00%)	0 (0.00%)
adenowirus	3 (8.82%)	3 (8.82%)
hMPV	0 (0.00%)	0 (0.00%)
enterovirus	11 (32.35%)	4 (47.06%)
rhinovirus	12 (35.29%)	9 (47.06%)
**Total**	**26 (76.47%)**	**34 (100%)**

a number of clinical samples tested positive with PCR;

b number of clinical samples tested positive with PCR and pre-amplified on HAE cultures.

**Table 3:** Detection of respiratory pathogens in clinical samples.

Patient	Detected pathogen [PCR^a^]	Detected pathogen [HAE cultures^b^]
1	rhinowirus, betacoronavirus, enterovirus, adenovirus	N.T.^d^
2	rhinowirus, enterovirus	N.T.
3	influenza A	N.T.
4	N.D.^c^	enterovirus, alphacoronavirus
5	N.D.	enterovirus, betacoronavirus, rhinovirus
6	N.D.	enterovirus, alphacoronavirus
7	rhinowirus, enterovirus	N.T.
8	N.D.	enterovirus, parainfluenza 2, rhinovirus
9	rhinowirus	N.T.
10	rhinowirus, parainfluenza 3, influenza B	N.T.
11	enterovirus	N.T.
12	rhinowirus, adenovirus	N.T.
13	parainfluenza 1,enterovirus	N.T.
14	rhinowirus, adenovirus	N.T.
15	N.D.	rhinovirus
16	N.D.	enterovirus, parainfluenza 2
17	rhinowirus, alphacoronavirus	N.T.
18	rhinowirus	N.T.
19	Enterovirus	N.T.
20	Enterovirus	N.T.
21	rhinowirus	N.T.
22	betacoronavirus	N.T.
23	Enterovirus	N.T.
24	rhinowirus, Influenza A	N.T.
25	N.D.	parainfluenza 1
26	influenza A, adenovirus	N.T.
27	Enterovirus	N.T.
28	influenza A	N.T.
29	N.D.	alphacoronavirus, rhinovirus
30	influenza A, adenovirus	N.T.
31	rhinowirus, parainfluenza 3	N.T.
32	Enterovirus	N.T.
33	parainfluenza 3, enterovirus	N.T.
34	influenza A, influenza B	N.T.

a clinical samples tested with PCR;

b clinical samples pre-amplified on HAE cultures, tested with PCR;

c N.D.: not detected;

d N.T.: not tested.

### Improved detection of pathogens following the HAE culture

**Figure 6:**
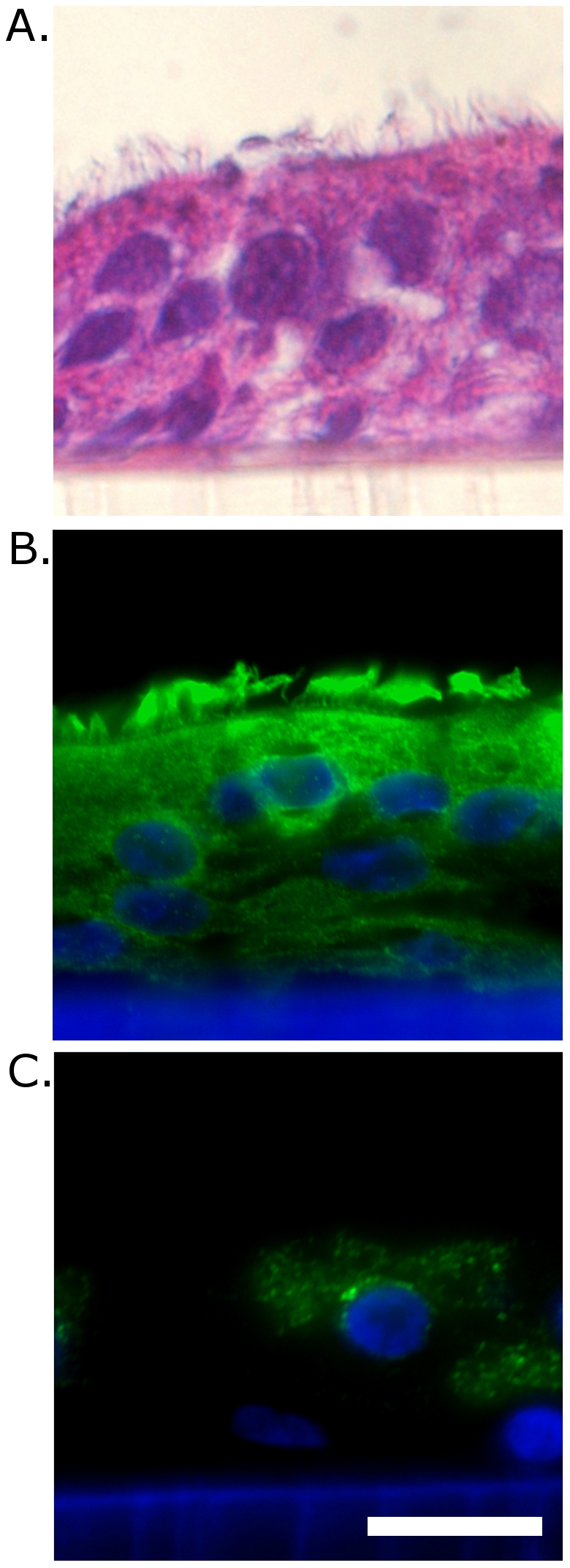
HAE culture architecture. A) Haematoxylin and eosin staining B) β-tubulin and nuclear staining, C) mucin 5AC and nuclear staining. Images were obtained employing fluorescence microscope at 600× magnification; Scale bar: 25 µm.

**Figure 7:**
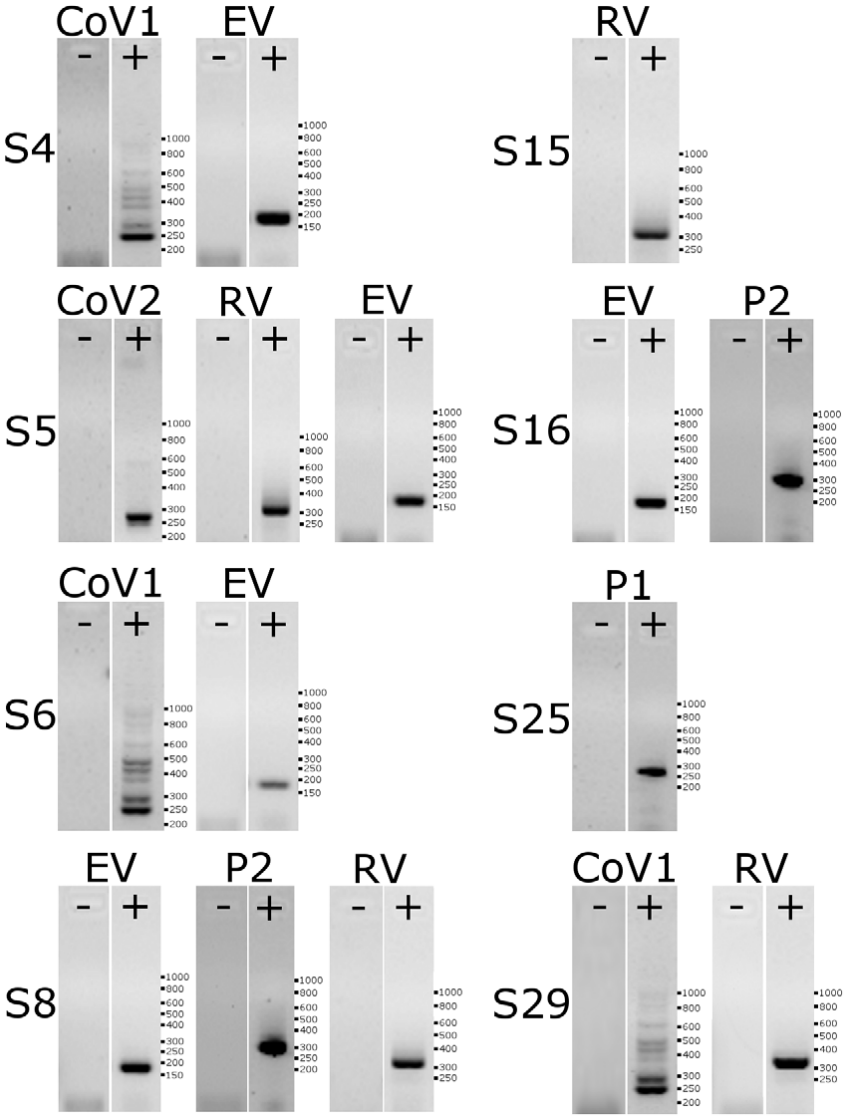
Pre-amplification on HAE cultures improves detection of viral pathogens. RT-PCR analysis of clinical specimens tested negatively for all known pathogens (denoted as “−”) and pre-amplified on HAE cultures (denoted as “+”). “S” denotes the sample number. CoV_1: alphacoronaviruses; P1: parainfluenza virus type 1; P2: parainfluenza virus type 2; EV: enteroviruses; RV: rhinoviruses. GeneRuler 50–1000 bp DNA (Fermentas) marker was used as a size marker. Analysis was performed on 1.5% agarose gel.

As mentioned previously, an etiological agent cannot be found in ∼30% of clinical specimens from patients suffering from respiratory tract illnesses [8], [9], [10], [11], [12]. A similar detection rate was observed in the current study; therefore, fully-differentiated HAE cultures (**Figure 6**) were inoculated with clinical specimens that remained undiagnosed by standard assays. A total of 8 samples were tested. Apical washes were collected following incubation for 72 h or 144 h post-inoculation at 37°C. RNA or DNA from these samples was isolated and submitted for nested PCR analysis. Combining the sensitivity of the developed diagnostic sets with the potency of HAE cultures resulted in viral pathogens being detected in all the tested samples (**Table 3** **,** **Figure 7**).

## Discussion

At present, the most sensitive and specific methods for detection, typing and identification of viruses are based on molecular biology techniques. Numerous molecular assays have been described, though *in silico* analysis reveals that the majority of these methods are based on the sequence of only a single viral isolate, and that their performance in laboratory settings is limited (data not shown). In the current study, a broad and sensitive panel of assays based on a two-step nested PCR was developed for the detection of a majority of human respiratory viruses. Selected primer sets, designed based on complete GenBank information, are complementary to 95–100% of the sequences of archival and contemporary viral strains. Such an approach limits the influence of intra–species sequence variability and facilitates the detection of contemporary and (most likely) newly emerging strains. The developed assays may thus be considered not only as detection techniques but also as a basic tool for virus discovery. The main disadvantage of developed tests is that detection is limited to viruses that belong to specific viral families/subfamilies, and they cannot be used for all viruses. Furthermore, the diversity of viral species in combination with their low load in clinical samples may result in false-negatives [41]. This particular feature makes these assays inferior to sequence-independent methods (e.g. VIDISCA), which can detect unknown pathogens [23], [42]. Then again, assays based on degenerated primers offer far higher sensitivity than those based on sequence-independent detection techniques.

The detection of unknown viral pathogens in respiratory clinical material is difficult using sequence-independent virus discovery methods, as the low viral load and high background signal from cellular nucleic acids frequently hinders detection. Until now, sequence independent virus discovery techniques were mostly used for virus culture supernatants (as they contain highly concentrated viral nucleic acids) [23], [43], or for the discovery of previously unknown DNA viruses [44], [45], [46]. Thus far, no study has identified a novel human respiratory RNA virus using sequence independent amplification techniques. Even though several improvements have been proposed [47], [48], [49], the sensitivity of these methods is inferior to that of nested or real-time PCR.

In the current study, detection of viral pathogens was possible in ∼76% of clinical samples, leaving ∼24% of cases undiagnosed. Such a detection rate is consistent with that in previously published reports [8], [9], [10], [11], [12]. To test whether it was possible to type the infectious agents in the remaining samples, molecular assays were developed and coupled with *ex vivo* cell culture techniques based on fully differentiated human airway epithelium.

HAE cultures constitute a semi-natural environment for the replication of viral pathogens, and pre-amplification of viruses contained in otherwise negative clinical samples resulted in the detection of viral agent(s) in 100% of cases. This observation is compliant with the fact that the HAE model is superior to any other culture system in terms of amplifying respiratory viruses. HAE cultures allow replication of majority of the respiratory viral species, including those that cannot be cultured in cell lines.

Again, one may question whether using HAE cultures will bias the results, as it is known that human metapneumovirus, for example, does not replicate in such a model (unpublished observation). This assumption is undoubtedly correct, although as mentioned above, this culture model is superior for the majority of respiratory viruses and is unrivalled in terms of virus discovery.

The system developed in the present study for the detection of respiratory pathogens represents a two-stage screening system, in which clinical samples can be tested for known viruses, and samples that remain undiagnosed may be amplified in *ex vivo* HAE cultures. Such amplification may result in enhanced detection of viruses in clinical samples during the second round testing with universal primers and, eventually, in the identification of obstacles that hinder the detection of pathogens (e.g. strain variability, presence of PCR inhibitors). Furthermore, these cultures may serve as a source of input material for sequence-independent amplification, which require high virus yield and good signal/background ratios.

In conclusion, the present study describes a set of assays with sensitivity as low as 1–10 copies per reaction that can be used in a routine diagnostic laboratory. The obtained results clearly show that the evaluated tests are highly specific and sensitive. Thorough testing showed that the developed primer sets do not cross react with other respiratory pathogens and are able to detect viruses in a wide range of clinical samples. Further, HAE cultures can be used for pre-amplification of the signal contained in the sample and to facilitate detection of pathogens by standard PCR or sequence-independent methods. Based on these observations, one may hypothesize that the reported lack of an etiological agent in a proportion of patients suffering from respiratory disease may result not only from the presence of unknown viral species, but also from a lack of sensitivity of the available assays. Further studies, including VIDISCA analysis, will provide greater insights into the epidemiology of different strains and species, and may possibly allow the detection and identification of previously unknown viruses.

## Materials and Methods

### Virus preparation, titration and culture

Virus stocks were generated by infecting HeLa cells (ATCC: CCL-2) with adenovirus; RD cells (ATCC: CCL-136) with enterovirus; LLC-MK2 cells (ATCC: CCL-7; kind gift from Lia van der Hoek) with parainfluenza type 1, 2 and 3, HCoV-NL63, hMPV and hRSV; and MDCK.2 cells (ATCC: CCL-2936) with influenza A and B. In all the cases, cells were lysed after appearance of the cytopathic effect by two freeze-thawing cycles. The virus-containing fluid was aliquoted and stored at −80°C. A control cell lysate from mock-infected cells was prepared in the same manner as the virus stock.

Virus yield for all pathogens was assessed by titration on fully confluent susceptible cells, according to Reed and Muench formula [50]. Plates were incubated at 32°C or 37°C, and the cytopathic effect occurrence was scored using an inverted microscope.

Human coronavirus 229E, parainfluenza 1 and 2 viruses, and bocavirus originated directly from clinical samples processed in our laboratory. Stock samples containing influenza A virus, human echovirus 9, RSV, human parainfluenza 3 virus and human adenovirus were kindly provided by Marcel Muller; human coronavirus NL63 was a kind gift from Lia van der Hoek. Human metapneumovirus and human coronavirus HKU1 were kindly provided by Oliver Schildgen and Astrid Vabret, respectively. Influenza B virus was successfully isolated in our laboratory in the past.

### Human airway epithelium cultures

Human tracheobronchial epithelial cells were obtained from airway specimens resected from patients undergoing surgery under Silesian Center for Heart Diseases – approved protocols. This study was approved by the bioethical committee of the Medical University of Silesia in Katowice, Poland (approval no: KNW/0022/KB1/17/10 dated on 16.02.2010). A written informed consent was obtained from all patients. Primary cells detached from human bronchi and trachea with pronase E were expanded on collagen-coated (collagen type IV, Sigma aldrich) plastic in bronchial epithelial growth media (BEGM) to generate passage 1 cells and plated at density of 3×10^5^ cells per well on permeable Transwell inserts (6.5-mm-diameter; Corning Transwell-Clear) supports in BEGM. Cells were cultured at 37°C in presence of 5% CO_2_ until confluence. Human airway epithelium (HAE) cultures were generated by changing the media to Air Liquid Interface media (ALI) and provision of an air-liquid interface for 6 to 8 weeks to form well-differentiated, polarized cultures that resemble *in vivo* pseudostratified mucociliary epithelium. All procedures were performed as previously described [34].

### Histology and immunohistochemistry

For visualization of HAE structures, cultures on Transwell inserts were washed one time with 1× PBS and fixed with 400 µl of 4% paraformaldehyde in 1× PBS. Samples were incubated for 15 min at room temperature and membranes were detached from the plastic frame. Subsequently, membranes were washed with 1× PBS, dehydrated in a gradient of ethanol and xylene, paraffin embedded, sectioned (5 µm) and mounted on silanised glass slides. Prior to staining, slides were deparaffinized with xylene and rehydrated in ethanol.

Morphology of HAE cultures was visualized by means of standard haematoxylin and eosin method. For fluorescence microscopy, slides were incubated consecutively in 0.2% Tween 20 (20 min, room temperature) and 2% bovine serum albumin (1 h, room temperature). Subsequently, for immunolocalisation of β5-Tubulin and mucin 5AC samples were incubated with mouse monoclonal anti-recombinant β5-Tubulin (Santa Cruz; dilution 1∶100) and mouse monoclonal anti-recombinant Mucin 5AC, Santa Cruz; dilution 1∶50), respectively. Goat anti-mouse IgG (H+L) Alexa Fluor 488 (Invitrogen; dilution 1∶200;) was used as a secondary antibody. Nuclear staining was performed using Hoechst 33258 (Sigma-Aldrich; 0.5 µg/mL). Sections were examined with fluorescent microscope (Nicon Eclipse Ti).

### Clinical samples

Clinical samples from adult patients with asthma exacerbations were submitted to the Jagiellonian University Medical College, Krakow, Poland. Samples (nasal washings obtained by the lavages) were collected as a part of standard procedure for diagnosis of respiratory pathogens. This study was approved by the Bioethical Committee of the Jagiellonian University in Krakow, Poland (approval no: KBET/68/B/2008 dated on 25.09.2008; extended until 30.11.2012). A written informed consent was obtained from all patients. Following the collection samples were stored at −80°C.

### Design of primers

In order to design primers that would recognize all strains of a given virus, irrespectively of natural variability, all genomic sequences available via GenBank were obtained. Sequences were organized with BioEdit software and further aligned with ClustalW script (http://www.ebi.ac.uk/). For influenza A sequence alignments, sets containing 500 sequences were generated, due to limitations of the alignment software. Conserved sites in sequence sets were identified with BioEdit and inspected by eye. For larger data sets, a visual basic script was developed to identify the presence of sites conserved in more than 95% of analyzed genomes. All positions were further verified in terms of primer quality and specificity with Blast server (http://blast.ncbi.nlm.nih.gov/Blast.cgi) and with OligoAnalyzer 3.1 (http://eu.idtdna.com/analyzer/applications/oligoanalyzer/). In order to correct for strain variability, 3′ part of the primer was designed at the conserved site, while 5′ part was degenerated, if required.

### Nucleic acid extraction

RNA from clinical samples and viral culture supernatants was extracted using RNA mini kit (A&A Biotechnology, Gdynia, Poland), according to manufacturer's protocol. DNA was isolated using Viral DNA/RNA mini kit (A&A Biotechnology, Gdynia, Poland), according to manufacturer's instructions. Isolated nucleic acids were stored at −80°C.

### Generation of plasmid-based positive controls

In order to develop positive controls that can be easily quantified, a set of plasmids containing DNA fragment targeted by dedicated primers was prepared. Briefly, isolated RNA was reverse transcribed with High Capacity cDNA Reverse Transcription (Applied Biosystems), according to manufacturer instructions, using 15 µl of the previously extracted RNA. Isolated DNA from samples containing DNA viruses was used as input for PCR without any processing. First PCR reaction was conducted as described below and resulting mixtures were layered on 1.5% agarose gel. Following gel separation of DNA fragments, bands corresponding in size to the expected products were extracted from the gel and total DNA was purified with Gel-out kit (A&A Biotechnology, Gdynia, Poland). DNA concentration was further assessed spectrophotometrically and cloned with InsTAclone PCR Cloning kit (Fermentas) into pTZ57R/T vector. After amplification in DH5α *E. Coli* and plasmid isolation with Plasmid mini kit (A&A Biotechnology, Gdynia, Poland), identity of cloned cDNA fragments was confirmed with DNA sequencing. Subsequently, plasmid DNA was linearized with EcoRI restriction enzyme (Fermentas), separated with gel electrophoresis and purified from the gel, as described above. DNA concentration was assessed spectrophotometrically and by gel analysis and number of copies per mL was calculated. Generated plasmids were further used as positive controls for PCR reaction.

### Reverse transcription and PCR reactions

Isolated nucleic acids (reverse transcribed for RNA viruses) were used as an input material for two-round PCR reaction with selected primers. Amplification was carried out in a total volume of 10 µl with DreamTaq PCR Master Mix (Fermentas), in presence of forward and reverse primers (1^st^ PCR: 200 nM each; 2^nd^ PCR: 600 nM each) and template DNA (1^st^ PCR: 2 µl; 2^nd^ PCR: 2 µl). The first PCR (touch-down PCR protocol) cycling conditions included initial denaturation for 3 minutes at 95°C followed by 13 cycles of 20 sec at 95°C, 30 sec at 68°C (which was decreased by 1°C per cycle), 40 sec at 72°C. A further 27 cycles of 20 sec at 95°C, 30 sec at 56°C, 40 sec at 72°C then followed and 5 min at 72°C for the final elongation.

For the nested PCR, reaction was prepared in the same manner, with 2.0 µl of the 1^st^ PCR mixture as a template. The cycling conditions for the nested PCR consisted of an initial denaturation for 2 min at 95°C followed by 35 cycles of 20 sec at 95°C, 30 sec at 56°C, 40 sec at 72°C, and an additional 5 min at 72°C for final elongation. Following the nested PCR the whole sample (10 µl) was analyzed on a 2.5% (influenza A assay) or 1.5% (other assays) agarose gel and visualized with ethidium bromide.

### Evaluation of assay sensitivity

In order to determine the sensitivity of each assay, serial dilutions of linearized positive control plasmids were used in concentrations ranging from 10^0^–10^5^ copies per reaction. PCR and nested PCR were conducted as described above. Analysis was performed with standard gel electrophoresis. Further, assays were evaluated with cell culture material or clinical samples positive for HCoV-NL63, HCoV-229E, HCoV-HKU1, RSV, influenza A and B viruses, parainfluenza 1, 2, 3 viruses, human bocavirus, adenovirus type 4, human metapneumovirus and human echovirus 9. Virus stocks were diluted in culture media or clinical samples tested negatively for all known pathogens, including sputum, bronchioalveolar lavage, and nasal lavages.

### Evaluation of assay specificity

Viral nucleic acids were isolated as described above and reverse transcribed where appropriate. Concentrated viral samples were tested with all primer sets by two-round PCR and validity of assays was evaluated. Viruses used in this study include HCoV-229E, HCoV-NL63, HCoV-HKU1, influenza A and B viruses, parainfluenza 1, 2, 3 viruses, bocavirus, RSV, human adenovirus, human metapneumovirus and human echovirus 9.
